# Multiresolution dendritic cell algorithm for network anomaly detection

**DOI:** 10.7717/peerj-cs.749

**Published:** 2021-10-19

**Authors:** David Limon-Cantu, Vicente Alarcon-Aquino

**Affiliations:** Department of Computing, Electronics and Mechatronics, Universidad de las Americas Puebla, San Andres Cholula, Puebla, Mexico

**Keywords:** Network anomaly detection, Intruder detection systems, Artificial immune systems, Machine learning, Wavelet transforms, Wavelets, Dendritic cell algorithm

## Abstract

Anomaly detection in computer networks is a complex task that requires the distinction of normality and anomaly. Network attack detection in information systems is a constant challenge in computer security research, as information systems provide essential services for enterprises and individuals. The consequences of these attacks could be the access, disclosure, or modification of information, as well as denial of computer services and resources. Intrusion Detection Systems (IDS) are developed as solutions to detect anomalous behavior, such as denial of service, and backdoors. The proposed model was inspired by the behavior of dendritic cells and their interactions with the human immune system, known as Dendritic Cell Algorithm (DCA), and combines the use of Multiresolution Analysis (MRA) Maximal Overlap Discrete Wavelet Transform (MODWT), as well as the segmented deterministic DCA approach (S-dDCA). The proposed approach is a binary classifier that aims to analyze a time-frequency representation of time-series data obtained from high-level network features, in order to classify data as normal or anomalous. The MODWT was used to extract the approximations of two input signal categories at different levels of decomposition, and are used as processing elements for the multi resolution DCA. The model was evaluated using the NSL-KDD, UNSW-NB15, CIC-IDS2017 and CSE-CIC-IDS2018 datasets, containing contemporary network traffic and attacks. The proposed MRA S-dDCA model achieved an accuracy of 97.37%, 99.97%, 99.56%, and 99.75% for the tested datasets, respectively. Comparisons with the DCA and state-of-the-art approaches for network anomaly detection are presented. The proposed approach was able to surpass state-of-the-art approaches with UNSW-NB15 and CSECIC-IDS2018 datasets, whereas the results obtained with the NSL-KDD and CIC-IDS2017 datasets are competitive with machine learning approaches.

## Introduction

Security threat detection in information systems is an ever-evolving challenge for computer security. Research in this field has increased in relevance as information systems provide essential services for enterprises and individuals. Computer security threats are adverse or harmful events targeted to a computer system resource through passive (to learn or use information without affecting system resources) or active (to alter a system resources or affect a system’s normal operation) attacks, often exploiting vulnerabilities found in the target system. Anomaly detection refers to the problem of finding unexpected behavior, these are often known as anomalies, outliers or discordant observations (Chandola, Banerjee & Kumar, 2009), and are usually patterns not conforming with a notion of normal behavior. The detection of anomalous patterns consists on defining a region represented as normal behavior, and any element distant from such region is determined as anomalous. This distinction is achieved through several methods including searching, signature-based, anomaly-based, feature learning, and feature reduction. Environments changing over time can make the normal behavior not relevant and increase incorrect classifications, whereas certain observations tend to be similar to others, causing confusion in detecting anomalies.

Intrusion Detection Systems (IDS) aim to solve anomaly detection by analyzing computer networks and systems through monitoring and analysis. This is performed with tools such as machine learning algorithms and signature based detection, to generate alerts based on the status of the observed resources. IDS can be classified into two broad groups, namely Network Intrusion Detection Systems (NIDS) and Host Based Intrusion Detection Systems (HIDS). NIDS are IDS whose main purpose is to analyze network communications, find anomalies or predict incoming attacks. HIDS are, on the other hand, specific purpose IDS whose objective is to protect a specific computer system. This is commonly done through resource usage analysis of different elements available in an operating system environment, such as file access, process execution, and outgoing and incoming communications.

The human body protects itself against anomalies with a complex system, known as immune system. Some of the observed characteristics of this system are noise tolerance, distribution, self-organization, non-centralized control and enhanced memory (Murphy et al., 2008). The study and understanding of these features are prime candidates for creating computer models capable of anomaly detection. Since the 1990s, there have been an increasing interest in the research and development of computational bio-inspired immune models in several areas, such as optimization, computer security, and pattern recognition. The ongoing battle between security researchers and security threats calls for the development of models able to overcome contemporary attacks. The Human Immune System (HIS) is a multi-layered and highly distributed system made up of different cell types and organs, and has evolved to provide effective protection and regulation to viruses and infections. The HIS is one of the most important systems in the human body. It is plausible to achieve similar results in computer systems by mimicking the characteristics of the HIS (Pamukov, 2017; Rauf, 2018). The HIS is a set of biological elements aimed to protect an organism against disease, and is mainly composed of three layers, namely physical, innate and adaptive.

The physical layer is the first defense against harmful or unknown molecular structures, known as antigens, and protects human body cells and tissues as a response mechanism against disease. The physical layer is comprised of skin, gastrointestinal tract and blood barriers and provides the most immediate defense mechanism. The first layer of protection may be compromised, for example, by a cut or skin burning. The second HIS layer are the innate and adaptive immune systems, and provide further protection and response against invading organisms. The second immune system layer is comprised of specialized cells. Innate immune system cells include macrophage, natural killer, neutrophil, basophil, eosinophil, mast and dendritic. The adaptive immune system develops the ability to recognize specific pathogens, and includes lymphocytes.

Macrophages detect and kill foreign and unhealthy cells by engulfing them, also alerting the rest of the immune system. Additionally, they clean the system of dead or necrotic cells. Natural killer cells also have the task of killing. However, they are specially focused on several types of tumors and microbial infections. They also interact with other immune cells. Neutrophills have multiple functions, and aim to neutralize threats; similar to natural killer cells, with additional control mechanisms such as blocking and disabling antigens. Basophils, eosinophils and mast cells provide similar protection to allergic and inflammatory reactions, as well as to combat infections, and are present in many tissues throughout the body. Dendritic Cells (DC) are Antigen Presenting Cells (APC). Their main role is to collect signals emitted by other cells and ingest residuals caused by normal (programmed cell death) and anomalous behavior (cell damage), to provide the adaptive immune system cells (such as lymphocytes) with antigen definitions. The results favor the proliferation of defense cells able to react to specific threats and kick-start the adaptive immune response. Lymphocytes are white blood cells able to recognize antigens and generate neutralizing antibodies (proteins that bind to foreign antigens). They are divided in T-cells (cell-mediated immunity matured in the thymus), and B-cells (humoral immunity cells matured in the bone marrow).

The Danger Theory model (Matzinger, 1994) is mainly centered in specific interactions of certain immune cells and alert signals as a response mechanism to defend the host system. Alert signals denote when a cell or a tissue is experiencing regular or abnormal behavior, such as expected or unexpected cell death, stress or inflammation caused by antigens. The danger theory proposes that part of the immune system is able to suppress an immune response, as it has been observed, the HIS does not always respond and eliminate all non-self sources, mainly allergic reactions, bacteria colonies inside human intestines and autoimmune diseases, among others (Garrett, 2005; Murphy et al., 2008). We can associate the HIS with IDS through anomaly detection.

Dendritic cells can be seen as detectors, as well as mediators in the human immune system. The Dendritic Cell Algorithm (DCA) is a population-based binary classifier designed for anomaly detection. The algorithm is inspired by the function of DCs, which are a part of the innate immune system. It incorporates danger theory principles by proposing an abstract model of DCs and its interactions with molecular information, as to induce an appropiate immune response towards possible threats.

The main contribution of this research is a biologically inspired NIDS based on the DCA (Gu, Greensmith & Aickelin, 2013). The proposed approach (MRA S-dDCA) incorporates the use of multiresolution analysis (MRA), as well as a segmentation approach to the deterministic DCA (S-dDCA). This model aims to tackle three contemporary issues, namely feature selection, classification performance, and proposing solutions to DCA related issues. Conversely from machine learning approaches for anomaly detection, the DCA does not have a training phase, shortening the algorithm process. The inclusion of decision trees in the classification process aims to improve the model performance, in comparison to the commonly used classification threshold method. The implementation of the deterministic DCA performs linear calculations, having a low weight in computation and providing an alternative to solve the intrusion detection problem. A comparison with the deterministic DCA, as well as different machine learning techniques is performed by using publicly available datasets, namely NSL-KDD, UNSW-NB15, CIC-IDS2017 and CSE-CIC-IDS2018.

### Related work

For the purposes of IDS research, network attacks are considered anomalous behavior. IDS models can be classified depending on the way they learn to discriminate between anomalies, namely supervised and unsupervised. The former refers to learning by using manually labeled observations to improve correct detection. The latter refers to determining if the observed data is normal, without prior knowledge. Network communications are observed by collecting and processing data generated as part of communication interfaces and devices. Network features are commonly categorized as low-level and high-level. Low level features include raw packet data, payload, session, and traffic data collected by network devices, such as routers. High-level features can provide further information about network communications and status, such as flow data, logs, and statistics. More elaborated features can be generated by using specialized tools such as *Argus* and *Zeek*. These tools process low-level and high-level data to generate additional features. Three contemporary approach categories are presented, namely *machine learning*, *metaheuristic*, and *artificial immune systems*.

*Machine learning algorithms* can be divided in two broad groups, namely *deep* and *shallow* (Liu & Lang, 2019). The main discerning factor between the two groups are the way features from observations are represented. Deep learning techniques (Potluri, Ahmed & Diedrich, 2018; Hou et al., 2020; Huang & Lei, 2020; Zhang, Yu & Li, 2018) can learn feature representations beyond the provided features and create *hyper-parameters*, or internal representations of the processed data by using abstraction layers, thus the *depth*. *Shallow* learning techniques (Kuttranont et al., 2017; Jing & Chen, 2019), are characterized for their lack of *depth* in feature processing.

*Metaheuristic* methods for anomaly detection have been developed around several natural, as well as non-natural phenomena. Nature has developed efficient methods to achieve several tasks with a limited set of resources. These methods are commonly divided in *trajectory, population, natural and non-natural* (Abdel-Basset, Abdel-Fatah & Sangaiah, 2018). Contemporary methods have focused on the improvement of feature selection as part of anomaly detection models, such as Deep Neural Networks (DNN), Long-Short Term Memroy (LSTM), Deep Belief Networks (DBN) and Multi-Layer Perceptron(MLP) (Ghanem et al., 2021; Elmasry, Akbulut & Zaim, 2020). Natural (or bio-inspired methods) include Evolutionary Algorithms (EA), such as Genetic Algorithms (GA) and have been used to improve the problem search space, and used in tandem with Whale Optimization Algorithm (Tao, Sun & Sun, 2018), as well as a method for feature selection and parameter optimization to improve Support Vector Machine (SVM) efficiency (Vijayanand & Devaraj, 2020). Swarm inspired methods, such as Particule Swarm Optimization (PSO), have been used to optimize the weights of a Fast Learning Network (FLN) (Ali et al., 2018). Artificial Bee Colony (ABC) algorithm (Mazini, Shirazi & Mahdavi, 2019) has been used to perform classification in imbalanced datasets without relying on deep learning nor class balancing techniques. Non-natural phenomena, such as the Metaheuristic Association Scale (MAS) method has been implemented to perform feature optimization in the detection of Distributed Denial of Service (DDoS) attacks (Dasari et al., 2020).

*Artificial Immune Systems* are models inspired by the behavior of the HIS. Their aim is to imitate its biological counterpart favorable qualities, such as anomaly detection, noise resistance, distributed learning and non-central control (Tan, 2016). In comparison to other bioinspired models, such as GA, the immune system is sorely focused on the protection of its host system, and thus is an ideal inspiration for anomaly detection models. AIS have seen two generations of algorithms developed (Greensmith, Whitbrook & Aickelin, 2010). First generation models were designed around general abstractions of *traditional* immune models, such as negative selection (Belhadj Aissa, Guerroumi & Derhab, 2020), clonal selection (Lysenko, Bobrovnikova & Savenko, 2018), and immune networks (Shi et al., 2017).

In contrast, second generation models are designed using emerging immunology models, such as danger theory (Matzinger, 1994). The DCA is one of such second generation models. The algorithm is able to assess whether a group of observations, commonly network communications, is anomalous or normal through a detection and classification mechanism. The DCA algorithm evolution has been marked by three different contributions, starting with the *prototype* DCA (Greensmith, Aickelin & Cayzer, 2005), followed by a more elaborated version using stochastic elements, known as *stochastic* DCA (Greensmith, Aickelin & Twycross, 2006a). This proposal has been further developed as the *deterministic* DCA (Greensmith & Gale, 2017). Stochastic based methods (Farzadnia, Shirazi & Nowroozi, 2020) simulate the behavior of DC by using random data sampling and processing derived from network features. Deterministic approaches reduce the use of random elements and focus on predictability of artificial DC behavior and data processing. These approaches have incorporated additional mechanisms to improve detection capabilities, such as fuzzy logic (Elisa et al., 2019), and biological to artificial feature mapping (Elisa, Chao & Yang, 2020).

The approach in Sharma & Tiwari (2018) performs anomaly detection using high-level network features, by implementing a modified probabilistic DCA. The danger theory inspired model in Alaparthy & Morgera (2018) employs several immune inspired mechanisms to perform network attack detection in a wireless sensor network using low-level features. The approach in Almasalmeh, Saidi & Trabelsi (2019) compares the performance of the deterministic and stochastic DCA and is aimed to detect malicious port scanning in the transmission control protocol by using a combination of both low-level and high-level features. The work in Elisa, Chao & Yang (2020) performs classification using high-level network features, and proposes a modification of the DCA in order to improve classification in large datasets, while also reducing model overfitting. The approach employs GA as an optimization mechanism.

The rest of this paper is organized as follows. “Background” presents a biological to computational concept mapping for the proposed model, as well as the background for the proposed model. “Proposed Model” describes the proposed anomaly detection model. “Results” deals with dataset descriptions used for testing, model parameters, and numeric results. A comparison with state-of-the-art approaches is also presented. “Discussion” presents a discussion of the obtained results. “Conclusions” concludes this paper and presents future work.

## Background

**Figure 1:**
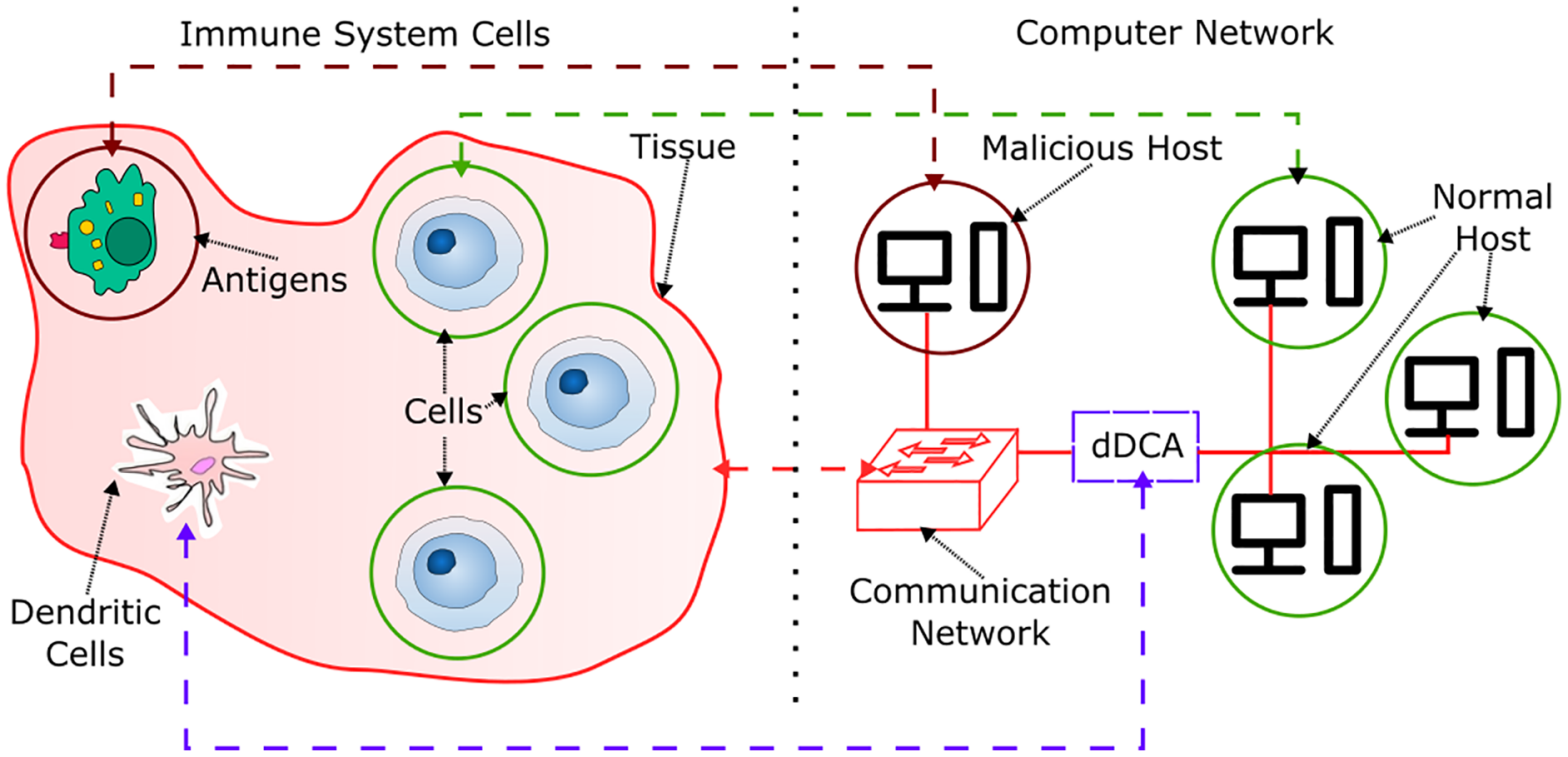
HIS and dDCA analogy.

In order to provide context for the development of a DCA inspired IDS, an analogy between biological and computational context is presented in Fig. 1. In the HIS, cells are contained in tissues, which are themselves a set of cells, these are part of the communication environment in the body, and can be related to communication networks in computer systems, such as network switches and network servers. Antigens can relate to malicious hosts, where a host is a network capable computer that aims to perform malicious activity to non-harmful or normal hosts. Normal behavior can be characterized as a set of activities and patterns in a communication network, where their presence is a signal of expected behavior in the network, and are originated from normal hosts. However, there can be moments in time where normal activities can behave unexpectedly. These kinds of anomalous behavior are not harmful. As the active defense mechanism in the human body, the HIS is comprised by a set of cells capable of detecting, disabling and destroying harmful or malfunctioning cells and antigens.

### The dendritic cell algorithm

Artificial immune systems were developed by observing the human immune system behavior, and are modeled after one or more interactions of immune cells and organs. The HIS aims to provide protection and defense mechanisms against invading agents, such as bacteria, parasites and virus. The development of IDS have drawn inspiration from immune mechanisms, such as negative selection, clonal selection, immune networks, and danger theory. The danger theory is one of the prominent HIS models and have provided inspiration to develop an algorithm based on the behavior of dendritic cells, known as the DCA. The DCA algorithm evolution has been marked by three different contributions, starting with the *prototype DCA* (Greensmith, Aickelin & Cayzer, 2005), followed by a more elaborated version using stochastic elements (Greensmith, Twycross & Aickelin, 2006b) and further developed as the *deterministic DCA* (dDCA) (Greensmith & Aickelin, 2008, Greensmith & Gale, 2017). The different specifications of the algorithm provide a similar framework; however, they differ in key aspects that determine its behavior. As the focus of this research is to develop a model based on the dDCA, any subsequent mentions of the DCA are related to its deterministic version. The DCA has three phases, namely feature selection, detection and context assessment, and classification.

### Feature selection

The danger theory model (Matzinger, 1994) is mainly centered in the interactions of signals emitted by cells and antigens. These signals denote when a cell or a tissue is experiencing regular or abnormal behavior, such as expected or unexpected cell death, stresss, inflammation, or anomalous processes caused by malfunctioning cells. The signals are categorized in three groups, namely Pathogen Associated Molecular Pattern (PAMP), Safe Signals (SS), and Danger Signals (DS). The deterministic adaptation of the DCA (Greensmith, Twycross & Aickelin, 2006b) requires input data to be represented as two signal categories, namely DS and SS, a categorical value that identifies each data instance, and a unique identifier for each data instance. The preprocessing and initialization phase assigns a feature, or a set of features, from the original dataset to each of the required signal categories. The proposed approach relies on feature-class multual information for signal categorization, followed by an average feature transformation for each category, to determine the features with the most influence (Elisa et al., 2019; Witten et al., 2017). Given two dataset features, represented as discrete random variables *F* and *C*, mutual information *I*(*F*;*C*) is the amount of information that a random variable *C* gives about *F*, as shown in Eq. (1),

(1) $$I(F;C) = \sum\limits_{f \in F} \sum\limits_{c \in C} p(f,c)log\left(\displaystyle{{p(f,c)} \over {p(f)p(c)}}\right)$$

where *p*(*f*) and *p*(*c*) are the marginal probabilities, and *p*(*f*,*c*) represents the joint probability mass function of discrete random variables *F*, *C*, as shown in Eq. (2),

(2) $$p(f,c) = P(C = c|F = f) \cdot P(F = f) = P(F = f|C = c) \cdot P(C = c)$$

where *P*(*C* = *c*|*F* = *f*) represents the probability of a sample of random variable *C*, given a different sample of random variable *F*. In order to categorize the selected features, Eq. (3) shows feature-class mutual information between each attribute and class. Mutual information of the feature *F*_*i*_ whose behavior is considered *normal F*^*s*^_*i*_, as well as *anomalous F*^*d*^_*i*_, and dataset classes *C* is obtained. The difference of absolute value between both results is calculated. A difference greater than zero indicates mutual information between normal behavior present in feature *F*_*i*_ and dataset classes is greater than that of anomalous behavior, thus the feature is categorized as SS. Conversely, if the attribute is lower or equal to zero, the feature is categorized as DS. Each of the available features *F*_*i*_ in the dataset is compared using this approach.

(3) $$FC({F_i}) = |I(F_i^s,C)| - |I(F_i^d,C)|$$

After feature selection and categorization, *DS* and *SS* signal categories are generated, where ${\vec F_{d \in D}}$ and ${\vec F_{s \in S}}$ represent the feature vectors that have been selected for each signal category using Eq. (3). This process is detailed in Eqs. (4) and (5), where *count*([*D*,*S*]) represents the number of features selected for each signal category.

(4) $$DS = \displaystyle{{\sum\nolimits_{d \in D} {{\vec F}_d}} \over {count(D)}}$$

(5) $$SS = \displaystyle{{\sum\nolimits_{s \in S} {{\vec F}_s}} \over {count(S)}}$$

#### Wavelet transform

The wavelet transform is a mathematical tool used to measure variations of a signal in the time-frequecy domain at different time-frequency resolutions. In comparison to Fourier analysis, wavelet analysis consists on decomposing a signal into shifted and scaled variations of rapidly decaying wave-like oscillating functions known as *wavelets*. The decomposition process can be achieved through an orthogonal set of components known as scaling (approximation) *ϕ* and wavelet (detail) functions *ψ*. These functions need to constitute orthonormal bases for Lebesque space ${L^2}({\rm {\mathbb R}})$ (Mallat, 1989). This transformation allows to isolate high-frequency low-duration, as well as low-frequency large-duration phenomena. Several Wavelet families have been developed, such as *Daubechies*, nearly symmetrical *Symlets* and *Coiflet* (Daubechies, 1992).

Multiresolution analysis (MRA) is the process of expressing a signal in terms of lower-resolution approximations and details to succesively create higher resolution versions, until the original signal is recreated. In wavelet analysis, the maximum amount of MRA levels that can be decomposed are 2^*J*^, where *J* = *log*_2_(*N*) and *N* is the finite signal (or function) length. A decomposed signal in each level *j* is a decimated version of length 2^*J*^. A finite time signal, such as *DS* or *SS* signal categories [*DS*,*SS*](*t*), where *t* represents an arbitrary point in time, is expressed as a recursive relationship for each level of decomposition (Alarcon-Aquino & Barria, 2009), where each level *j* ∈ *J*, *j* ≤ *J* contains lower approximation and detail coefficients; the sub-spaces comprising the decomposed signal have to meet certain criteria (Mallat, 1989). The Discrete Wavelet Transform (DWT) is given in Eq. (6),

(6) $$[DS,SS](t) = \sum\limits_{n \in {\rm {\mathbb Z}}} {c_{J,n}}{\phi _{J,n}}(t) + \sum\limits_{j = J}^\infty \sum\limits_{n \in {\rm {\mathbb Z}}} {d_{j,n}}{\psi _{j,n}}(t)$$

where *n* represents a translation of signal [*DS*,*SS*](*t*) in the integer domain ${\rm {\mathbb Z}}$, for detail, or approximation coefficients (*ψ*_*j*,*n*_ and *ϕ*_*j*,*n*_(*t*) respectively), *J* denotes the maximum decomposition level, *n* is the filter length for approximation and detail coefficients, *j* represents a decomposition level, such that *j* < *J*, and wavelet *ψ*(*t*) and scaling functions *ϕ*(*t*), are a family of orthonormal bases (Burrus et al., 1998). The signal [*DS*,*SS*(*t*)] is decomposed in details *d*_*j*,*n*_ and approximations *c*_*j*,*n*_ to form a multiresolution analysis. There exists a recursive relationship between the coefficients at successive levels of decomposition. Eqs. (7) and (8), Daubechies (1992) show the details *d*_*j*,*n*_ and approximations *c*_*j*,*n*_ for the DWT as recursive functions of filter coefficients, where *g*_*l*_ represents scaling filters and *h*_*l*_ are the wavelet filter coefficients, and *l* is a displacement factor related to the length of the wavelet or scaling filter.

(7) $${c_{j,n}} = \sum\limits_{l \in {\rm {\mathbb Z}}} {g_l}{c_{j - 1,2n - l}}$$

(8) $${d_{j,n}} = \sum\limits_{l \in {\rm {\mathbb Z}}} {h_l}{c_{j - 1,2n - l}}$$

The DWT requires the signal sample size to be a multiple of 2^*J*^, due to the decimation process at each level of decomposition. This limitation can introduce time ambiguities in the decomposed signals. The coefficients of the DWT can introduce a *blurring* effect in the signal due to its compactly supported Conjugate Quadrature Filters (CQF) (Daubechies, 1988). The Maximal Overlap Discrete Wavelet Transform (MODWT), also known as non-decimated, stationary, translation or time invariant DWT (Nason & Silverman, 1995; Liang & Parks, 1996; Pesquet, Krim & Carfantan, 1996) is a higly redundant and non-orthonormal transform that performs a decomposition process similar to MRA DWT, while having several favorable properties. Among the most notable is the amount of computations per decomposition level *j* < *J*, where the MODWT requires a computational complexity of *O*(*Nlog*_2_*N*), as opposed to *O*(*N*) for the DWT, thus having a greater computational cost. Another important property is the fact that the MODWT prevents signal down-sampling, a property that is necessary to perform analysis using the DCA, as well as allowing a signal of arbitrary length to be decomposed (Percival & Walden, 2006).

The MODWT wavelet and scaling filters are related to the DWT filters, such that ${\tilde h_l},{\tilde g_l}$ are re-scaled versions that conserve signal energy (Percival & Walden, 2000) given by ${\tilde h_l} \equiv {h_l}/\sqrt 2 ,{\tilde g_l} \equiv {g_l}/\sqrt 2$. This implies $\sum\nolimits_{l = 0}^{L - 1} \tilde g_l^2 = \sum\nolimits_{l = 0}^{L - 1} \tilde h_l^2 = \displaystyle{1 \over 2}$, where *L* denotes the filter length, and the filters must satisfy specific conditions (Percival & Walden, 2000). The MODWT MRA decomposition details $d_{j,n}^{(O)}$ and approximations $c_{j,n}^{(O)}$ in Eqs. (9) and (10) can be generated by a pyramid algorithm and are obtained as circular filter operations of a time series. As time localization in time series analysis for anomaly detection is necessary to precisely identify the presence of anomalies, MODWT allows to analyze the different decomposition levels of a finite time series, without the decimation effect of DWT, while conserving important characteristics of MRA as to provide an accurate representation of a signal at different time-frequencies.

(9) $$c_{j,n}^{(O)} = \sum\limits_{l = 0}^{L - 1} {\tilde g_l}c_{j - 1,(n - {2^{j - 1}}l)\text{mod}\ {\it N}}^{(O)}$$

(10) $$d_{j,n}^{(O)} = \sum\limits_{l = 0}^{L - 1} {\tilde h_l}c_{j - 1,(n - {2^{j - 1}}l)\text{mod}\ {\it N}}^{(O)}$$

The MODWT is used to decompose signals [*DS*,*SS*](*t*) while preserving energy, and preventing the signal downsampling present in the DWT, in order to perform analysis in the time domain, while also providing approximation $c_{j,n}^{(O)}$ and details $d_{j,n}^{(O)}$ at different decomposition levels. Eq. (11) shows the MODWT MRA process for [*DS*,*SS*] signal categories.

(11) $$[DS,SS](t) = \sum\limits_{n \in {\rm {\mathbb Z}}} c_{J,n}^{(O)}{\phi _{J,n}}(t) + \sum\limits_{j = J}^\infty \sum\limits_{n \in {\rm {\mathbb Z}}} d_{j,n}^{(O)}{\psi _{j,n}}(t)$$

### Detection and context assessment

The DCA incorporates the use of two intermediate signals, known as Co-stimulatory Molecule Signal (CSM) (Greensmith, Aickelin & Cayzer, 2005), and $\hat k$ (Greensmith & Aickelin, 2008). These are defined in Eqs. (12) and (13) respectively.

(12) $$CS{M_p}(t + 1) = \left\{ {\matrix{ {CS{M_p}(t) + (SS(t + 1) + DS(t + 1)),} {{\rm if}\ CS{M_p}(t) \le m{t_p}} \cr {0,} {\rm otherwise} \cr } } \right.$$

In Eq. (12), *CSM*_*p*_(*t* + 1) represents the signal concentration at time *t* + 1, akin to the costimulatory signal value in the DCA approach (Greensmith & Aickelin, 2008), where *CSM*_*p*_(0) = 0, and *p* represents a DC in the population. [*SS*,*DS*](*t* + 1) are the signal values at time *t* = {1, 2, …, *N*}. The role of *CSM*_*p*_(*t*) is to limit the time a DC at any time *t* in the population *p* spends on antigen sampling by imitating a cell’s lifespan. When a DC has exceeded maturation threshold, defined as *mt*_*p*_, it migrates to a separate DC pool, namely the migrated pool, and no longer samples antigens. The DC that migrates is replaced with a newborn cell whose *CSM* and $\hat k$ values are 0. The deterministic DCA employs ${\hat k_p}$ (Greensmith & Aickelin, 2008) to reflect the magnitude of signal concentration in a cell. This is shown in Eq. (13), where ${\hat k_p}(0) = 0$, *p* represents a DC in the population, [*SS*,*DS*](*t* + 1) are signal values at time *t* = {1, 2, …, *N*}, and *mt*_*p*_ is the migration threshold for the cell *p* in the population (Gu, Greensmith & Aickelin, 2013).

(13) $${\hat k_p}(t + 1) = \left\{ {\matrix{ {{{\hat k}_p}(t) + (DS(t + 1) - 2\,SS(t + 1)),}  {{\rm if}\ CS{M_p}(t) \le m{t_p}} \cr {0,} {\rm otherwise} \cr } } \right.$$

The context assessment phase consists on adding the signal concentration ${\hat k_r}$ of each migrated cell *r* to the antigen repository *k*(*α*), where *α* is the antigen category. This repository contains the sum of ${\hat k_r}$ that have sampled antigen *α*, divided by the times the antigen was sampled by a DC in the population (Greensmith & Aickelin, 2008), as shown in Eq. (14), where *r* represents a migrated cell in the migrated cell population *R*, and *r* ∈ *R*.

(14) $$k(\alpha ) = \displaystyle{{\sum\nolimits_{r \in R} ({{\hat k}_r}(\alpha ))} \over {count(\alpha )}}$$

#### Segmentation approach

The segmented dDCA, or S-dDCA (Gu, Greensmith & Aickelin, 2009a; Gu, Greensmith & Aickelin, 2013) was introduced as an alternative approach to the dDCA. Segmentation was introduced as a granular signal analysis approach, conversely from the coarse approach of the deterministic DCA, where given a dataset of size *N*, all samples are processed before performing classification. The segmented DCA shares all phases of the deterministic DCA. The main premise of this approach is to perform detection and context assessment using a reduced amount of data instances, thus the dataset needs to be partitioned into $m = \textstyle{N \over M}$ *segments*, where *M* is the desired segment size, *N* is the dataset size, and *m* is the segment count. Antigen categories *α* are also modified to represent individual observations. The finer-grained approach allows the algorithm to perform the detection and context assessment in a non-sequential manner, at the expense of performing context assessment *m* times. Computational complexity of the model can be linear (Gu, Greensmith & Aickelin, 2013), as DC population *p* and segment size *M* are usually significantly lower than *N*. Segmentation approach has not seen widespread adoption in the DCA research field, as contemporary proposals tend to solve other DCA challenges, such as pre-processing (Chelly & Elouedi, 2013a, Chelly & Elouedi, 2013b, Chelly & Elouedi, 2016) and feature tuning (Chelly & Elouedi, 2011).

### Classification

The classification phase consists on evaluating the antigen repository *k*(*α*). The deterministic DCA employs the use of the *T*_*k*_ classification threshold (Greensmith & Aickelin, 2008), where any *k*(*α*) greater than a given threshold is classified as anomalous. This threshold is commonly set as a user-defined parameter, or derived from observations obtained in the detection phase. Using a linear classification threshold is known to have issues (Gu et al., 2011), as it may not properly separate normal data instances using *k*(*α*). The use of a decision tree classifier removes the use of such classification threshold.

A Decision Tree (DT) is a supervised learning model commonly used for classification and regression tasks. The main objective of a DT is to build a model based on (simple) decision rules that are derived from data predictors. A decision tree is built in a sequential manner, where a set of simple tests are combined logically. For example, comparing a numeric value against a threshold or a specific range, or comparing a categorical value against a set of possible categorical values. As an observation is compared against the set of rules generated by a DT, the observation is determined as belonging to the most frequent class present in that *region* (Hastie, Tibshirani & Friedman, 2009).

The objective of a decision tree is to partition the space given by a set of *features* or *predictors* by using a set of rules. A partition is generated in order to split the feature space into several regions, known as *branches* or *nodes*. This process is performed until a stopping criteria is met. When such criteria occurs, the final splits are used to determine the class of the observations, these final nodes are known as *leafs*. The process of generating a decision tree is to find a feasible strategy to determine a split criteria, by using a set of predictors or features, belonging to a set of observations. The general strategy used by most decision tree algorithms, such as *Classification and Regression Tree* (CART), is known as the *Hunt’s algorithm*. This algorithm consists on a recursive process that generates partitions by measuring the amount of elements that belong to the same class, also known as *impurity*. The objective is to generate subsets that reduce the impurity measure. Some favorable characteristic of decision trees are low computational complexity for prediction, not requiring large amounts of observation to generate a model, and transparency (as generated rules can be visualized). Decision trees are also known to overfit. In order to solve this, several constraints and optimization features have been developed, such as pruning, sample number minimum for each leaf node, and maximum tree depth (Kotsiantis, 2013).

## Proposed Model

**Figure 2:**
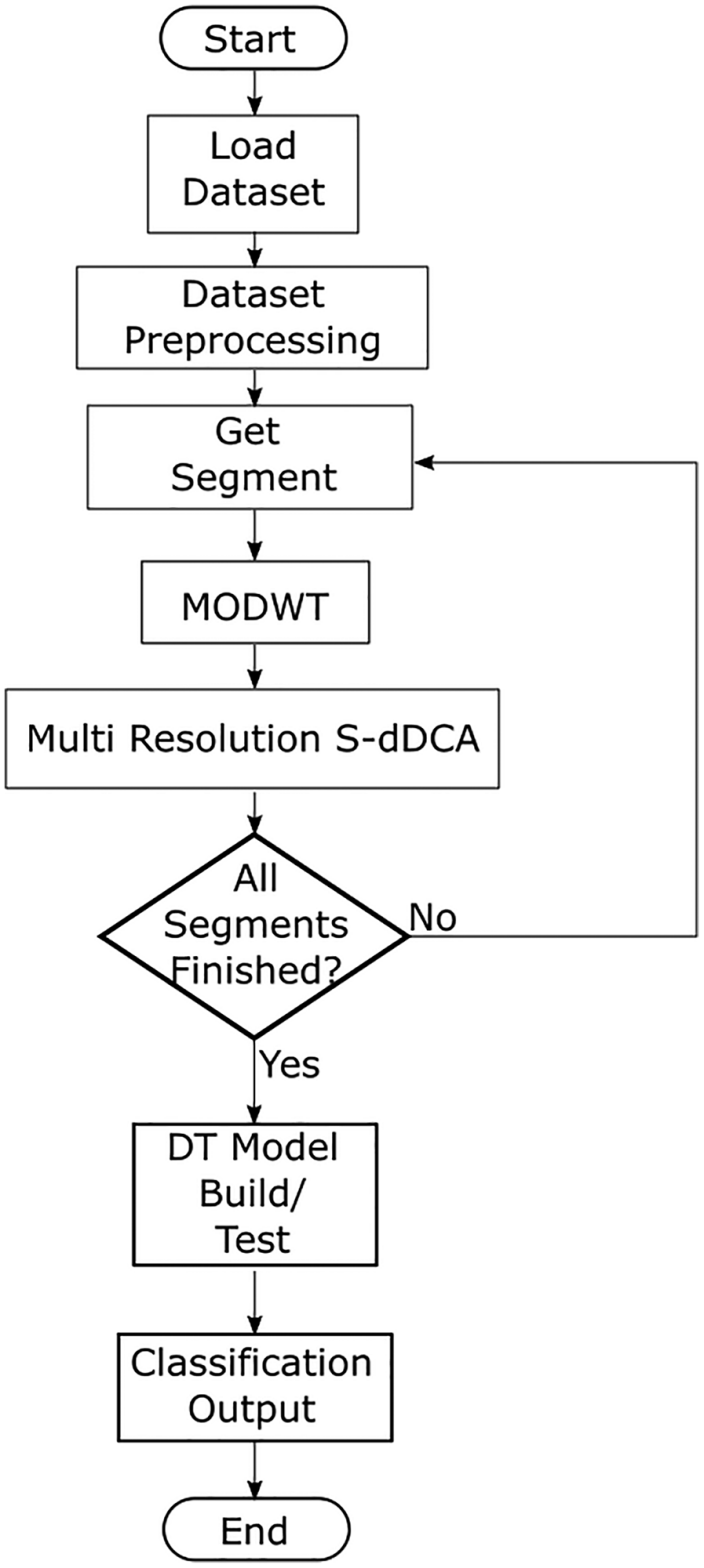
Proposed multiresolution segmented dDCA model.

The proposed model aims to incorporate a the segmented dDCA as a granular classification approach, as well as using MODWT decomposed versions of *SS* and *DS* signal categories as part of the detection and context assessment. The proposed model flow diagram is presented in Fig. 2. After loading a dataset, features are selected using feature-class mutual information (Gu, Greensmith & Aickelin, 2013), as proposed in Eqs. (1)–(3). The most relevant features related to the normal and attack classes are obtained, and can be user limited. A generalized approach is used for feature aggregation, where the selected features are averaged (all features have the same weight), to generate the signal categories used as inputs for the S-dDCA algorithm (Elisa et al., 2019), as shown in Eqs. (4) and (5). Additionally, the processed dataset is comprised of a label for each data instance, where labels such as origin port, destination port and protocol are concatenated. The dataset is divided into *m* sequential segments; each segment is used to perform MODWT using wavelet *w*, and performs up to *J* transform levels, where *J* > 0, *J* <= *log*_2_*M*, and *M* is the segment size. The multiresolution approach aims to incorporate the decomposed signal at *J* levels to the context assessment of the DCA, where each DC in the population uses a decomposition level as input for each signal category. Eqs. (15) and (16) show the modified detection and context assessment phase, where *SS*(*t* + 1), *DS*(*t* + 1) is replaced with the corresponding decomposition details obtained using MDOWT. The DC population corresponds to MRA decomposition levels, such that *p* = *j*, and *j* ≤ *J*. Wavelet details of *SS* and *DS* signals decomposed up to *j*^*th*^ level are given as $ss_{j,t + 1}^{(O)}$ and $ds_{j,t + 1}^{(O)}$, respectively.

(15) $$CS{M_j}(t + 1) = \left\{ {\matrix{ {CS{M_j}(t) + (ss_{j,t + 1}^{(O)} + ds_{j,t + 1}^{(O)}),}  {{\rm if}\ CS{M_j}(t) \le m{t_j}} \cr {0,}  {\rm otherwise} \cr } } \right.$$

(16) $${\hat k_j}(t + 1) = \left\{ {\matrix{ {{{\hat k}_j}(t) + (ds_{j,t + 1}^{(O)} - 2 \cdot ss_{j,t + 1}^{(O)}),}  {{\rm if}\ CS{M_j}(t) \le m{t_j}} \cr {0,}  {\rm otherwise} \cr } } \right.$$

Once all data segments have been processed, a DT model is built using the antigen repository *k*(*α*), the signal energy for each decomposition level, and a categorical value for each data instance containing information from the communication source, or destination, such as port number. Each processed segment contributes to the antigen repository in an additive manner, and works independent from each other. Signal energy (Burrus et al., 1998) may contain variations when anomalies occur at different decomposition levels (Du et al., 2018), and is obtained for each signal category and segment at decomposition level *j* ≤ *J*.

**Figure 3:**
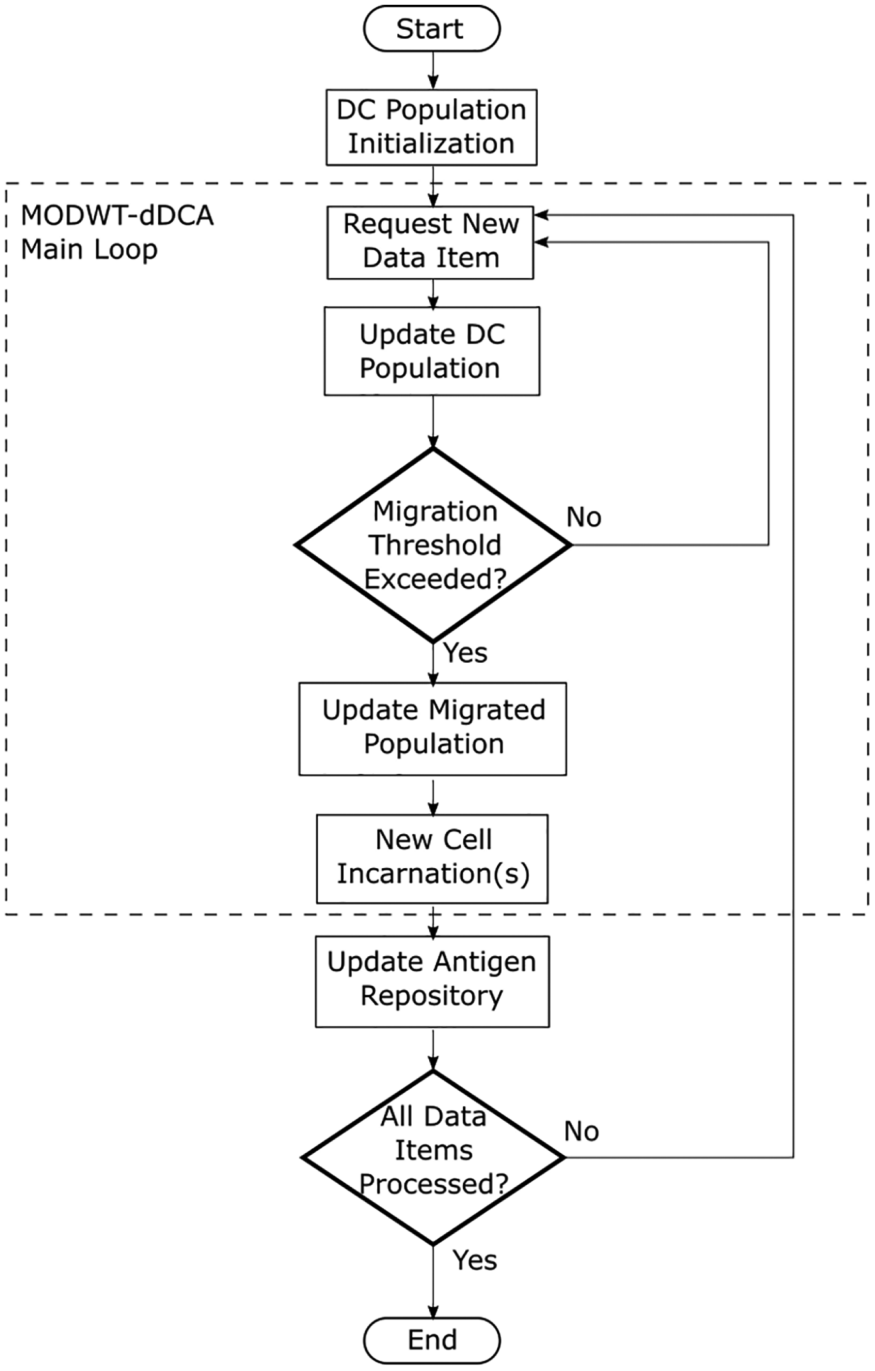
Dendritic cell algorithm flow diagram.

Additionally, Fig. 3 describes the multiresolution dDCA flow diagram. This process is executed for each segment *m*. The main loop is initialized as the cells in the population are assigned a migration threshold. The migration threshold *mt*_*j*_ is described as a uniform distribution with the range [0, 1]. As a new data item is requested, the decomposed signal for *DS* and *SS* at *j* ≤ *J* decomposition level is provided. Each cell in the DC receives its corresponding decomposition level, as proposed in Eqs. (15) and (16). This implies the DC population can have up to *J* <= *log*_2_*M* DCs, and *M* is the segment size. Detection and context assessment is performed for each DC in the population. When any DC has met the migration threshold, it transfers to the migrated DC population, and a new DC is created to take its place. Finally, the antigen repository is updated. This process is repeated until no more data items are left to process for the segment.

## Results

The proposed model was developed using the MATLAB R2021a environment in a computer running the Linux operating system with an Intel Core i7 8,700 CPU and 64.0 GB of RAM. The testing was performed using the NSL-KDD, UNSW-NB15, CIC-IDS2017, and CSE-CIC-IDS2018 datasets. The proposed model performs binary classification. All attack categories present in the datasets are considered anomalies and are labeled as one, normal behavior is labeled as 0. A confusion matrix is used to describe performance of the tested datasets. For a binary classifier, the confusion matrix consists of positive *P* and negative *N* classes. The positive class refers to any attack present in the dataset (*i.e.*, anomalies). The negative class refers to normal behavior. In order to generate a confusion matrix, the classified records are compared against the dataset true classes (*i.e.*, ground truth), the elements of a confusion matrix are detailed as follows,

- Correctly classified attacks are considered True Positives *TP*.
- When *TP* records are incorrectly classified, they are considered False Negatives *FN*.
- In the case of normal behavior, correctly classified records are known as True Negatives *TN*.
- Incorrectly classified normal records are known as False Positives *FP*.

Performance metrics are generated for further analysis and comparison. Accuracy refers to the ratio of correctly classified instances to the total tested instances, either attacks or normal behavior, and is given in Eq. (17).

(17) $$Accuracy = \displaystyle{{TP + TN} \over {TP + TN + FP + FN}}$$

Precision represents the proportion of correctly classified attacks and is given in Eq. (18).

(18) $$Precision = \displaystyle{{TP} \over {TP + FP}}$$

Similarly, recall (or true positive rate) represents the probability of attack detection, and is given in Eq. (19).

(19) $$Recall = \displaystyle{{TP} \over {TP + FN}}$$

The F1-Score given in Eq. (20) represents the balance between precision and recall, while omitting true normal behavior. Conversely from the balanced accuracy metric, the *F*_1_ score does not include negative classification metric *TP*. The use of this score allows to analyze the positive detection capabilities of a model, and is adequate to analyze anomaly or outlier detection models.

(20) $$F1 - Score = \displaystyle{{2*TP} \over {2*TP + FP + FN}}$$

The False Positive Rate *FPR* in Eq. (21) is given by the ratio between false positives and the total amount of actual normal behavior observations.

(21) $$FPR = \displaystyle{{FP} \over {FP + TN}}$$

Conversely, the False Negative Rate *FNR* measures the true positive miss-classification rate, and is given in Eq. (22).

(22) $$FNR = \displaystyle{{FN} \over {FN + TP}}$$

The False Discovery Rate *FDR* in Eq. (23) represents the ratio of false positives, to the total amount of elements classified as anomalies.

(23) $$FDR = \displaystyle{{FP} \over {FP + TP}}$$

### Dataset description

The UNSW-NB15 dataset is a publicly available dataset that contains over 100 GB of traffic (Moustafa, Creech & Slay, 2017). The raw network packets were created using the IXIA PerfectStorm tool and are a hybrid of modern real activities and synthetic contemporary anomalous behavior, with nine different attack types, namely Fuzzers, Analysis, Backdoors, DoS, Exploits, Generic, Reconnaissance, Shellcode and Worms, as well as normal traffic. The dataset is divided into train and test sets (Moustafa & Slay, 2016). The training set contains 175,341 records (119,341 anomalous and 56,000 normal). The testing set, conversely, contains 82,332 records (45,332 anomalous and 37,000 normal). Two tools (Argus and Bro-IDS) along with 12 algorithms were used to generate 49 features divided in flow features, content features, time features, basic features and additionally generated features.

The NSL-KDD (Tavallaee et al., 2009) is a publicly available dataset developed by the Canadian Institute for Cybersecurity. This dataset contains four attack types, namely Denial of Service (DoS), Probe, User to Root Attack (U2R) and Remote to Local Attack (R2L), and normal traffic; in total, 41 features for each connection were generated. The improved dataset version was created to solve two main problems of the KDD-99 dataset, namely the distribution of the attacks in the train and test sets, and the over-inclusion of Denial of Service (DoS) attack types, *neptune* and *smurf*, in the test dataset. This dataset omits redundant or duplicate records in the train and test sets, incorporates balancing of records for the train and tests sets, in order to avoid dataset sub-sampling, as well as to reduce computational time in model testing. The NSL-KDD dataset has the same features and attack types as KDD-99. The complete training dataset contains 125,973 records (58,630 anomalous and 67,343 normal). There is a reduced version of the train set (KDD + Train_20Percent) that contains a 20% subset of the training set. The full testing dataset contains 22,544 records (12,833 anomalous and 9,711 normal). Additionally, there exists a testing dataset that does not include records that were not validated by all 21 classifiers used to match the KDD-99 ground truth labels in the dataset creation (Tavallaee et al., 2009).

The CIC-IDS2017 and CSE-CIC-IDS2018 datasets (Sharafaldin, Lashkari & Ghorbani, 2018) were developed by the Canadian Institute of Cybersecurity (CIC). Additionally, the CSE-CIC-IDS2018 dataset (Sharafaldin, Lashkari & Ghorbani, 2018) was developed in collaboration with the national cryptologic agency of Canada, known as the Communications Security Establishment (CSE). The datasets were developed to provide IDS models with large volume publicly available datasets to test diverse contemporary network attacks. Specific criteria was used to create the datasets (Gharib et al., 2016), as it was identified to be necessary to build a reliable dataset.

The CIC-IDS2017 data was captured over the course of 5 days. The dataset topology consists of two different local networks connected through the internet, where there is an attack and a victim network. The captured packets were used to extract 80 network flow features by using the CICFlowMeter tool. The attacks present in the dataset include Brute Force, Denial of Service (DoS), Distributed Denial of Service (DDoS), Heartbleed, Web Attack, Infiltration, and Botnet. The dataset contains a total of 2,830,743 records (557,646 anomalous and 2,273,097 normal). A dataset for testing was created by sampling the original dataset while preserving attack order and attack type proportions. The resulting test dataset contains 13,963 anomalous records and 56,806 normal records.

The CSE-CIC-IDS2018 topology contains 420 machines and 30 servers, and consists of an attack subnet, as well as five subnets of victims. The attack network is connected to the victims through the Internet. Similar to the CIC-IDS2017 dataset, 80 flow features were extracted from the captured packets using the CICFlowMeter tool. The resulting dataset consists of 16,233,002 records (2,748,294 anomalous and 13,484,708 normal). In comparison to CIC-IDS2017, the executed attacks in the dataset are a comprehensive set of contemporary attacks over a larger network. Attacks include Brute Force, DoS, Web, Infiltration, Botnet, DDoS and PortScan. Similar to the CIC-IDS2017 dataset, a dataset for testing was created by sampling the original records, preserving attack order and attack proportions. The resulting dataset contains 13,683 anomalous records and 67,482 normal records.

The attack types found in the four proposed datasets, namely NSL-KDD, UNSW-NB15, CIC-IDS2017 and CSE-CIC-IDS2018, are commonly used methods to compromise a network security. Attacks such as DoS and Port Scan are present in all datasets, whereas DDoS, Heartbleed and Botnet attacks are present in the more recent CIC-IDS2017 and CSE-CIC-IDS2018. Although the NSL-KDD dataset was widely used as a benchmark, it does not contain contemporary network flows. In comparison, the UNSW-NB15 incorporated additional attacks. The CIC-IDS2017 and CSE-CIC-IDS2018 contain similar attack types at different network scales and complexity. The attack types for the presented datasets and a brief explanation are detailed in Table 1.

**Table 1:** Attack types and descriptions for NSL-KDD, UNSW-NB15, CIC-IDS2017 andCSE-CIC-IDS2018 datasets.

Type	Description	Dataset
Normal	Normal transaction data.	NSL-KDD, UNSW-NB15
Fuzzers	Attempting to cause a program or network to suspend by feeding it with randomly generated data.	UNSW-NB15
Analysis/Port scan	A series of port scan, spam and HTML file attacks.	UNSW-NB15, CIC-IDS2017, CSE-CIC-IDS2018
Backdoors	Technique to bypass security mechanisms stealthily.	UNSW-NB15
DoS	Malicious attempt to make a network resource unavailable by overwhelming its capacity to serve requests.	NSL-KDD, UNSW-NB15, CIC-IDS2017, CSE-CIC-IDS2018
DDoS	Multiple compromised systems flood the target system by generating network traffic, with the intent of depleting the bandwidth or resources of the targeted system.	CIC-IDS2017, CSE-CIC-IDS2018
Exploits/Infiltration attack	Leverage the knowledge of a system or software vulnerability by exploiting it to achieve unauthorized access to a system.	UNSW-NB15, CIC-IDS2017, CSE-CIC-IDS2018
Generic	A technique that works against all block ciphers (encryption method) without consideration of its structure.	UNSW-NB15
Reconnaissance	Attacks that aim to gather information about the network.	NSL-KDD, UNSW-NB15
Shellcode	Small piece of code used to exploit a software vulnerability.	UNSW-NB15
Worms	A piece of code that replicates itself in order to spread over the network, relaying on exploits to gain access.	UNSW-NB15
User to root attack (U2R)	The gains access to a regular account on the system, and exploits vulnerabilities to gain root access.	NSL-KDD
Remote to local attack (R2L)	An attacker without an account sends packets to a system to gain access as a user by exploiting vulnerabilities.	NSL-KDD
Brute force	An attack that attempts to gain access to restricted content by trial and error, commonly used to guess passwords, discover hidden content in web applications, among others.	CIC-IDS2017, CSE-CIC-IDS2018
Heartbleed	An attack that sends a malformed heartbeat request through the Open Secure Socket Layer (SSL) implementation of the Transport Layer Security (TLS) protocol.	CIC-IDS2017
Web attack	Attacks pertaining to web-based protocols, such as SQL Injection, Cross-Site Scripting (XSS), and brute force login attempts over HTTP.	CIC-IDS2017, CSE-CIC-IDS2018
Botnet	A large number of internet enabled devices used to perform various attacks.	CIC-IDS2017, CSE-CIC-IDS2018

### Model parameters

The proposed model has four configurable parameters, namely the number of features to be selected for each signal category *T* based on feature-class mutual information (Elisa et al., 2019), the segment size used by the S-dDCA *m*, the population size *p* and the wavelet used for the MODWT process *w*. The number of features was set to *T* = 5. As a result, five features were selected for each of the signal categories, namely DS and SS, for each tested dataset. The selected features are summarized in Tables 2 and 3. Each signal category is equal to the normalized average of its corresponding features, in the range from zero to one. A combination of categorical attributes (if present in each dataset) were used as part of the antigen repository, namely *protocol*, *service*, *state*, *source port* and *destination port*. Signal energy from each segment is also used as a feature for the antigen repository.

**Table 2:** Selected features for signal categorization, NSL-KDD and UNSW-NB15 datasets.

NSL-KDD			UNSW-NB15		
Feature	Signal category	*FC* (*F*_*i*_)	Feature	Signal category	*FC* (*F*_*i*_)
dst_host_count	SS	0.0913	spkts	SS	0.6454
dst_bytes	SS	0.0617	sttl	SS	0.5080
dst_host_same_src_port_rate	SS	0.0224	dmean	SS	0.4493
srv_diff_host_rate	SS	0.0146	dttl	SS	0.4148
src_bytes	SS	0.0142	dload	SS	0.4112
count	DS	−0.1056	smean	DS	−1.4737
dst_host_srv_rerror_rate	DS	−0.0423	ct_dst_src_ltm	DS	−0.4939
duration	DS	−0.0401	rate	DS	−0.4822
srv_count	DS	−0.0399	ct_srv_src	DS	−0.4775
dst_host_srv_serror_rate	DS	−0.0392	ct_srv_dst	DS	−0.4401

**Table 3:** Selected features for signal categorization, CIC-IDS2017 and CSE-CIC-IDS2018 datasets.

CIC-IDS2017			CSE-CIC-IDS2018		
Feature	Signal category	*FC* (*F*_*i*_)	Feature	Signal category	*FC* (*F*_*i*_)
FwdPacketLengthMin	SS	0.6787	PktLenMax	SS	0.6618
FlowBytess	SS	0.3624	PktLenStd	SS	0.0547
FwdPacketLengthMax	SS	0.3296	FwdActDataPkts	SS	0.0118
FwdURGFlags	SS	0.1520	BwdPSHFlags	SS	0
CWEFlagCount	SS	0.1520	BwdURGFlags	SS	0
TotalBackwardPackets	DS	−1.6132	InitBwdWinByts	DS	−2.0298
SubflowBwdPackets	DS	−1.6132	InitFwdWinByts	DS	−1.6018
BwdHeaderLength	DS	−1.1659	FwdPktLenMax	DS	−1.4341
TotalFwdPackets	DS	−0.8473	BwdHeaderLen	DS	−1.1041
SubflowFwdPackets	DS	−0.8473	PktSizeAvg	DS	−0.9627

The tested wavelets, segment sizes and population sizes are presented in Table 4. Segment sizes tested were 128, 256, 512, 1,024, 2,048, 4,096, 8,192, and 16,384. The DC population sizes *p* were 1, 2, …, *log*_2_*m*, where *m* refers to each segment size tested. The wavelet families used for testing the *w* parameter were *Daubechies, Symlet*, and *Coiflet*. For the daubechies wavelet family, the wavelet vanishing moments tested were 1 to 20. The amount of vanishing moments used to test the symlet wavelet family was 2 to 20, whereas the coiflet family vanishing moments tested was 1 to 5. Each wavelet was tested with all segment sizes and population sizes. The DT model parameters are resumed in Table 5, and was designed using the *fitctree* MATLAB model builder. The DT model parameters are presented as follows. Two predictor categories are assigned, namely *Normal* and *Anomalous*. Predictors used for the model are *k*(*α*), a categorical value that contains port and protocol information, as well as the energy of each segment and signal category, namely *DS* and *SS*. The penalty for miss-classification is set to 1, whereas exact values were used as feature split for the node generation in the classification tree. The tree does not contain a maximum depth for the training process. The maximum amount of categories for each split node is set to 10. All leaves that come from the same parent are merged, as long as the risk (or impurity) is greater or equal to the parent node. Minimum branch nodes is set to 10, whereas prior probability calculation is obtained from the analyzed dataset (empirical). As the last step, the DT model is tested with the processed segment data. The generated DT model is tested to classify normal and anomalous data, akin to the dDCA model. Test results are used to generate the confusion matrix as well as classification metrics.

**Table 4:** Assessed wavelets for the proposed model.

Wavelet w	Vanishing moments	Segment size *m*	Population size
Daubechies (db)	1, 2, …, 20	128, 256, 512, 1,024, 2,048, 4,096, 8,192, 16,384	1, 2, …, *log*_2_*m*
Symlet (sym)	2, 3, …, 20		
Coiflet (coif)	1, 2, 3, 4, 5		

**Table 5:** DT model parameters.

Parameter	Value
Predictor categories	Normal, Anomalous
Predictors	*k*(*α*)
Predictor split	Exact search
Miss-classification cost	1
Max. categories	10
Leaf merging	Yes
Min. branch nodes	10
Prior probabilities	Empirical

### Numeric results

The resulting performance metrics were obtained by testing the proposed model ten times for each proposed wavelet, vanishing moments, and population sizes using four datasets, namely NSL-KDD, UNSW-NB15, CIC-IDS2017, and CSE-CIC-IDS2018. The proposed model used a random uniform distribution for each DC migration threshold in each run. Testing datasets for the NSL-KDD and UNSW-NB15 were used to obtain the performance metrics. The datasets created for testing the CIC-IDS2017 and CSE-CIC-IDS2018 datasets were used to obtain the performance metrics. A comparison was performed with the three-signal dDCA approach without MRA (Gu, Greensmith & Aickelin, 2013), using mutual information maximization (Elisa et al., 2019), with the incorporation of DT to substitute the classification threshold *t*_*k*_ (Greensmith & Aickelin, 2008). This was done order to demonstrate the improvements provided by the segmentation approach, along with the use of MRA MODWT, as well as the two-signal approach in (Greensmith & Aickelin, 2008). The confusion matrix of the proposed approach for each tested dataset are presented in Tables 6–9.

**Table 6:** NSL-KDD confusion matrix.

Actual class	Predicted class	
	Positive	Negative
Positive	12,540	293
Negative	300	9,411

**Table 7:** UNSW-NB15 confusion matrix.

Actual class	Predicted class	
	Positive	Negative
Positive	45,326	6
Negative	12	36,988

**Table 8:** CIC-IDS2017 confusion matrix.

Actual class	Predicted class	
	Positive	Negative
Positive	13,772	159
Negative	149	56,612

**Table 9:** CSE-CIC-IDS2018 confusion matrix.

Actual class	Predicted class	
	Positive	Negative
Positive	13,541	137
Negative	64	66,931

The proposed model performance metrics are presented in Table 10. The best classification results obtained are highlighted in bold and were using *db1* and *sym2* for the NSL-KDD and the UNSW-NB15 datasets, respectively. The wavelets *sym5* and *db4* obtained the best results for the CIC-IDS2017 and CSE-CIC-IDS2018 datasets. The DC population size for the CIC-IDS2017 dataset was three, indicating that three decomposition levels were used as part of the detection and context assessment phase of the proposed model. For the remaining datasets, namely NSL-KDD, UNSW-NB15, and CSE-CIC-IDS2018, the DC population size was one. All the tested datasets achieved the best results with a segment size of 128. The proposed model was able to achieve an accuracy (Acc.) of 97.37%, 97.66% precision (Prec.), and 97.72% recall (Rec.), when tested with the NSL-KDD dataset. The UNSW-NB15 dataset achieved an accuracy of 99.97%, 99.98% precision, and 99.99% recall. The CIC-IDS2017 dataset achieved an accuracy of 99.56%, 98.86% precision, and 98.93% recall. The CSE-CIC-IDS2017 obtained an accuracy of 99.75%, 99% precision, and 99.53% recall. For the NSL-KDD, UNSW-NB15, CIC-IDS2017 and CSE-CIC-IDS2018 datasets respectively, the F1-Score (F1-S) obtained was 97.69%, 99.98%, 98.89%, and 99.26%. FPR achieved 3.09%, 0.03%, 0.28%, and 0.2%, whereas FDR achieved 2.34%, 0.03%, 1.14%, and 1%. FNR was 2.28%, 0.01%, 1.07%, and 0.47%. The dDCA + DT approach obtained a lower accuracy for all datasets tested, while showing an improvement in recall (99.20% and 100%) and FNR (0.8% and 0%) for the NSL-KDD and UNSW-NB15 datasets respectively. In comparison, the proposed MRA S-dDCA approach provided a considerable improvement in precision, F1-Score, FPR, FDR, and FNR for all datasets tested.

**Table 10:** Proposed model performance metrics.

Dataset	Model	Performance metrics (%)							Model parameters
		Acc.	Prec.	Rec.	F1-S.	FPR	FDR	FNR	
NSL-KDD	MRA S-dDCA	**97.37**	**97.66**	97.72	**97.69**	**3.09**	**2.34**	2.28	m = 128, p = 1, w = db1, T = 5
	dDCA + DT	93.29	88.93	**99.20**	93.79	12.86	11.07	**0.80**	p = 10, *mt^p^* = [0, 0.001]
UNSW-NB15	MRA S-dDCA	**99.97**	**99.98**	99.99	**99.98**	**0.03**	**0.03**	0.01	m = 128, p = 1, w = sym2, T = 5
	dDCA + DT	97.25	95.01	**100**	97.44	5.76	4.99	**0**	p = 10, *mt^p^* = [0, 0.001]
CIC-IDS2017	MRA S-dDCA	**99.56**	**98.86**	**98.93**	**98.89**	**0.28**	**1.14**	**1.07**	m = 128, p = 3, w = sym5, T = 5
	dDCA + DT	98.17	92.60	98	95.22	1.79	7.40	2	p = 10, *mt^p^* = [0, 0.001]
CSE-CIC-IDS2018	MRA S-dDCA	**99.75**	**99.00**	**99.53**	**99.26**	**0.20**	**1.00**	**0.47**	m = 128, p = 1, w = db4, T = 5
	dDCA + DT	92.30	56.05	97.46	71.17	8.26	43.95	2.54	p = 10, *mt^p^* = [0, 0.001]

**Note:**

The best classification results obtained are highlighted in bold and were using *db1* and *sym2* for the NSL-KDD and the UNSW-NB15 datasets respectively.

The main difference between the compared models, namely MRA S-dDCA and DCA + DT relates to the use of multiresolution analysis to analyze the algorithm signal categories, namely *SS* and *DS*. While the approaches such as (Zhou & Liang, 2021; Elisa et al., 2019) and dDCA + DT, process time series signals without additional signal processing after feature selection and signal categorization, the proposed approach performs time-frequency decomposition using multiresolution analysis. This approach allows the MRA S-dDCA to analyze the signal categories at different time-scale resolutions, while reducing the redundancy caused by performing antigen duplication when DC population *p* > 1. The segmentation approach allows the MRA decomposition process to be performed using a segment size *M*, while also allowing the S-dDCA approach to perform signal processing using a reduced number of samples, thus allowing the antigen repository to accumulate values that reduce dependability on larger dataset (or signal) sizes (Elisa, Yang & Naik, 2018).

**Figure 4:**
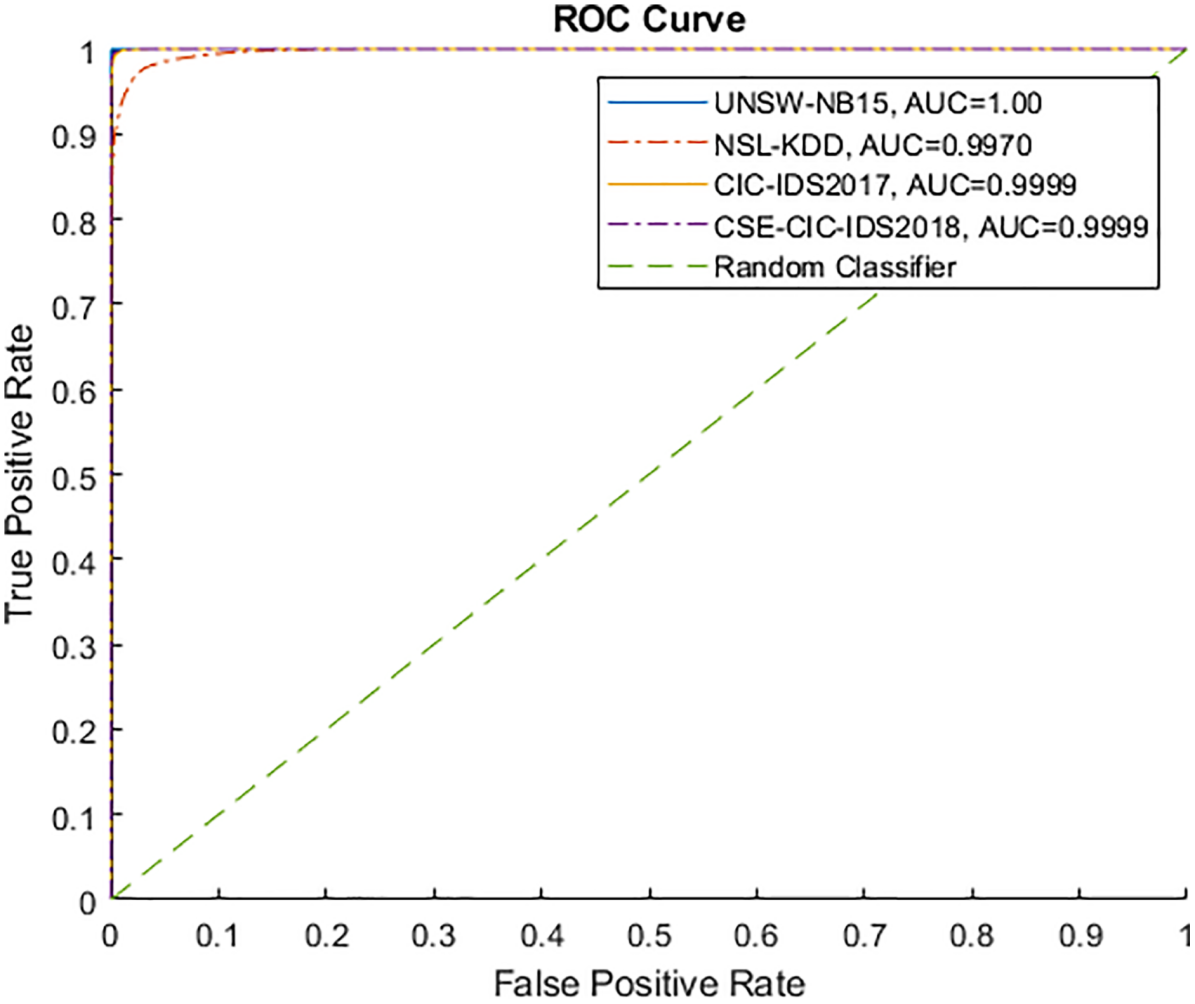
ROC curve for tested datasets.

**Figure 5:**
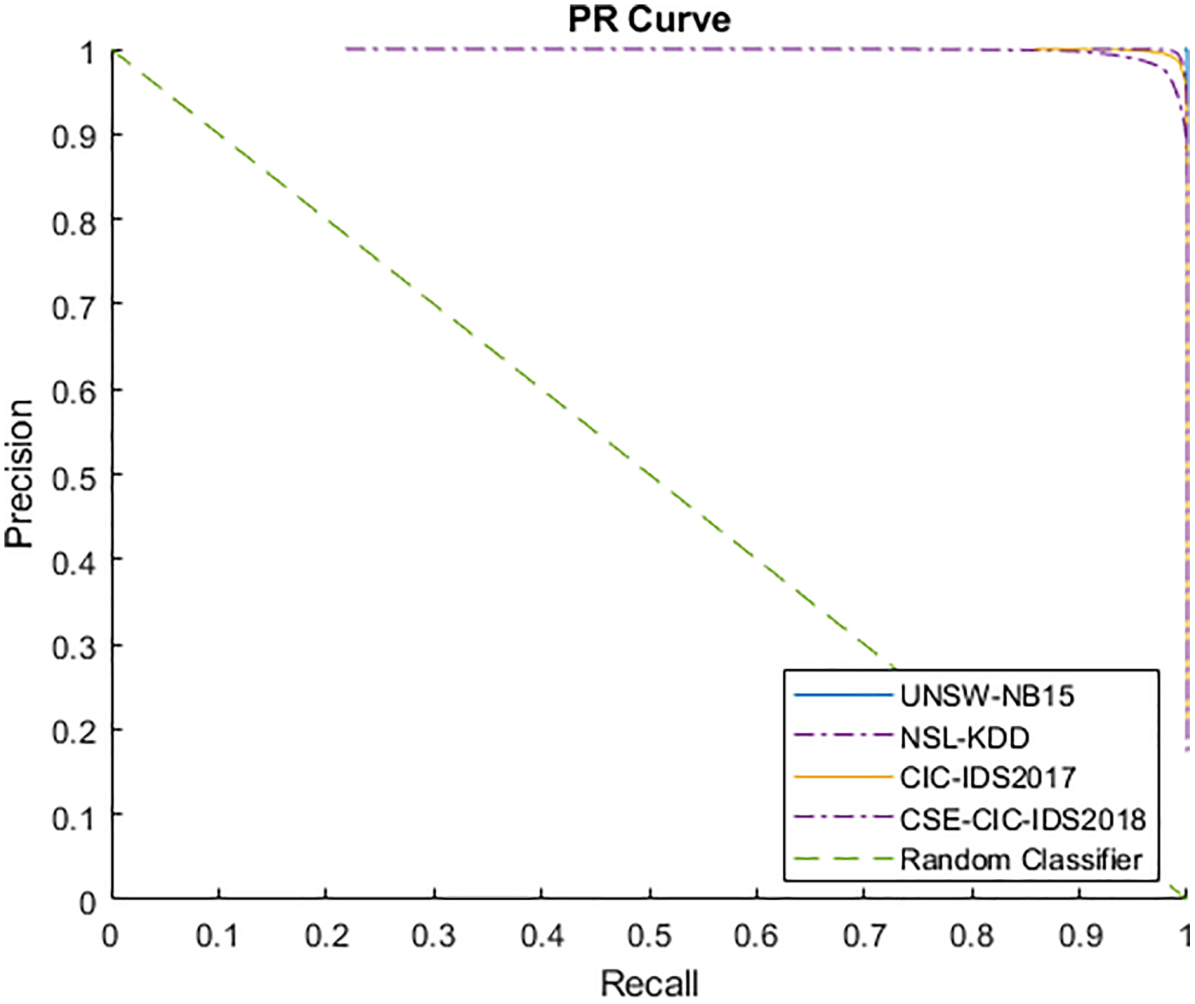
PR curve for tested datasets.

Figures 4 and 5 show the Receiver Operating Characteristic (ROC) and Precision-Recall (PR) curves for the tested datasets, namely NSL-KDD, UNSW-NB15, CIC-IDS2017, and CSE-CIC-IDS2018. The ROC curve shows the scores for the positive class (anomaly) resulting from the decision tree scores, based on the degree of certainty at the tree leafs. The resulting AUC curve for each datasets was 1.0 for the UNSW-NB15, 0.9970 for the NSL-KDD, and 0.9999 for the CIC-IDS2017 and CSE-CIC-IDS2018.

Contemporary state-of-the-art methods for binary classification are presented in Table 11. Accuracy (Acc.), precision (Prec.), recall (Rec.), F1-Score (F1-S.), False Positive Rate (FPR), False Discovery Rate (FDR), and False Negative Rate (FNR) are compared with the proposed model. The proposed model results are highlighted in bold and were obtained by evaluating the *test* datasets used in NSL-KDD and UNSW-NB15. The proposed *testing* datasets for the CIC-IDS2017 and CSE-CIC-IDS2018 were also used. The best accuracy result of 99.19% for the NSL-KDD dataset was obtained by the Adaboost Random Forest (Iwendi et al., 2020). The method using the eXtreme Gradient Boost Deep Neural Network (XGBoost-DNN) (Devan & Khare, 2020) provided the second best result, achieving a 97.60%, followed by 97.37% obtained by the proposed multiresolution DCA model (MRA S-dDCA). Other models compared include Convolutional Neural Network (CNN) (Belgrana et al., 2021) (95.54% accuracy), and Bidirectional Long Short-Term Memory Attention Mechanism with multiple Convolutional Layers (BAT-MC) (Su et al., 2020) (90.13% accuracy). When testing the UNSW-NB15 dataset, the MRA S-dDCA model obtained the best result, achieving 99.97% accuracy, followed by the Grasshopper Optimization Simulated Annealing algorithm (GOSA) (Dwivedi, Vardhan & Tripathi, 2020) achieving 98.96%, Bidirectional Gated Recurrent Unit with Hierarchical Attention Mechanism (GRU-HAM) (Liu et al., 2020) achieving 98.76%, Classifier Ensemble (Tama et al., 2020) achieving 92.45%, and Few Shot Learning (FSL) (Yu & Bian, 2020) achieving 92.0%. The best accuracy results for the CIC-IDS-2017 were obtained by Classifier Ensemble (Tama et al., 2020) with a 99.99%. The MRA S-dDCA obtained the second best result with a 99.56% accuracy, whereas the Spatial-Temporal Deep Learning on Communication Graphs (STDeepGraph) (Yao et al., 2019) obtained 99.40%. The Grasshopper Optimization Algorithm (GOA) obtained a 99.35%. The Heterogeneous Ensemble Learning Anomaly Detection (HELAD) obtained a F1-Score of 99.58%. For the CSE-CIC-IDS2018 dataset, the best accuracy result was obtained by the MRA S-dDCA with an accuracy of 99.75%. The Ensemble Learning and Feature Selection IDS (ELFS), as well as the Random Forest classifier (Fitni & Ramli, 2020) obtained an accuracy of 99.80%. The Decision Tree method (Fitni & Ramli, 2020) obtained 98.60%, whereas the Hybrid Convolutional Recurrent Neural Network-Based Network Intrusion Detection System (HCRNNIDS) achieved 97.75%. The precision, recall, and F1-Score reported in the XGBoost-DNN approach were surpassed by the MRA S-dDCA with 97.66%, 97.72%, and 97.69% respectively. The STDeepGraph approach in (Yao et al., 2019) surpassed the MRA S-dDCA in precision with a score of 99.30%, whereas the proposed model achieved better results in recall and FPR, with scores of 98.93% and 0.28% respectively. For the CSE-CIC-IDS2018, the MRA S-dDCA was able to surpass compared approaches in all tested metrics.

**Table 11:** Results comparisons with state-of-the-art approaches.

Dataset	Method	Acc. (%)	Prec. (%)	Rec. (%)	F1-S. (%)	FPR (%)	FDR (%)	FNR (%)
NSLKDD	Adaboost Random Forest (Iwendi et al., 2020)	99.19	99.45	98.86	99.16	0.50	0.55	1.14
	XGBoost-DNN (Devan & Khare, 2020)	97.60	97.00	97.00	97.00	–	–	–
	**MRA S-dDCA**	**97.37**	**97.66**	**97.72**	**97.69**	**3.09**	**2.34**	**2.28**
	CNN (Belgrana et al., 2021)	95.54	–	95.73	–	4.64	–	4.27
	BAT-MC (Su et al., 2020)	90.13	98.45	82.65	89.86	1.46	1.55	17.35
UNSWNB15	**MRA S-dDCA**	**99.97**	**99.98**	**99.99**	**99.98**	**0.03**	**0.03**	**0.01**
	GOSA (Dwivedi, Vardhan & Tripathi, 2020)	98.96%	–	–	–	0.084	–	1.15
	GRU-HAM (Liu et al., 2020)	98.76	99.35	98.94	–	–	–	–
	Classifier Ensemble (Tama et al., 2020)	92.45	88.70	87.79	88.25	5.33	11.30	12.21
	FSL (Yu & Bian, 2020)	92.00	–	–	–	8.01	–	7.89
CIC-IDS 2017	Classifier Ensemble (Tama et al., 2020)	99.99	99.54	100	99.77	0.01	0.46	0
	**MRA S-dDCA**	**99.56**	**98.86**	**98.93**	**98.89**	**0.28**	**1.14**	**1.07**
	STDeepGraph (Yao et al., 2019)	99.40	99.30	98.60	–	1.30	–	–
	GOA (Shukla, 2021)	99.35	–	–	–	0.05	–	–
	HELAD (Zhong et al., 2020)	–	99.58	99.58	99.58	2.15	–	–
CSE-CICIDS 2018	**MRA S-dDCA**	**99.75**	**99.00**	**99.53**	**99.26**	**0.20**	**1.00**	**0.47**
	ELFS (Fitni & Ramli, 2020)	98.80	98.80	97.10	97.90	–	–	–
	Random Forest (Fitni & Ramli, 2020)	98.80	98.70	97.00	97.80	–	–	–
	Decision Tree (Fitni & Ramli, 2020)	98.60	97.90	96.90	97.40	–	–	–
	HCRNNIDS (Khan, 2021)	97.75	96.33	97.12	97.60	2.5	–	3.00

**Note:**

The proposed model results are highlighted in bold.

## Discussion

The MRA S-dDCA performs context assessment by using a population of artificial DCs. Each element in the segment is sequentially processed. The segmented DCA performs this process at each segment with the aim of performing the dDCA process using a smaller data subset. A decomposition process is performed by using MODWT MRA. Detail coefficients at different decomposition levels are used as inputs for the proposed approach. This is performed to use high frequency components at different decomposition levels (or band-pass filters) as inputs for each DC in the population, and may provide insight related to network anomalies in the monitored traffic. This allows the algorithm to perform analysis using high frequency variations in the two signal categories used, while also avoiding data duplication such as the approach of antigen multiplication (Gu, Greensmith & Aickelin, 2008). The wavelets used to test the proposed approach were *Daubechies, Symlet*, and *Coiflet*. The use of energy for each signal category in any given segment is performed in order to provide the classification process with localized information about signal energy in each segment, as anomalies may cause a difference in signal energy at different decomposition levels. Once all segments have been processed, the anomaly metric coefficient *k*(*α*) is obtained. Finally, a decision tree model is generated based on the collected data in the antigen repository. The incorporation of MRA technique aims to further increase the model capabilities of performing analysis in the time-frequency space at different resolution levels, by using the MODWT, as well as to reduce the use of redundant observations that prior proposals have implemented as *antigen multiplication* (Gu, Greensmith & Aickelin, 2008; Oates et al., 2007; Gu et al., 2009b, Gu, Greensmith & Aickelin, 2013). The main drawback of this model resides on the dependence of segment size and DC migration threshold, where it is necessary to provide a segment where at least one cell migrates to the migrated cell population. If this does not occur, classification of any observation with no migrated cells may affect classification performance. As the proposed model is designed to process time series data, the presence of continuous attacks may induce more DCs in the population to migrate. Conversely, if there are not sufficient continuous attacks present, the DC migration rate may decrease. The effects of this can be the reduction of classification performance, such as the case with the NSL-KDD dataset, where the proposed model achieved third best result when comparing accuracy, and second best result when comparing precision, recall, and F1-Score. As the context detection phase performs linear operations, the model performance may also be affected when dealing with complex data from attacks that do not leave a significant footprint in the network traffic; this may be the case with the CIC-IDS2017 dataset, where an ensemble of classifiers was able to outperform the proposed model.

## Conclusions

Anomaly detection in computer networks analyze communications and aims to find unexpected or anomalous behavior that can be associated with attacks. These attacks aim to obtain protected data, exploit vulnerabilities found in computer systems, as well as to disable important systems, among other undesired behavior. The dendritic cell algorithm is an artificial immune system based on the behavior of dendritic cells, and is a population-based binary classifier designed for network anomaly detection. The proposed model was based on the danger theory. This paper proposed a feature selection approach, as well as a multiresolution based signal analysis mechanism and the segmented deterministic dendritic cell algorithm. Classification was performed using decision trees, and was evaluated using four publicly available datasets, namely UNSW-NB15, NSL-KDD, CIC-IDS2017 and CSE-CIC-IDS2018. The proposed model achieved an accuracy of 99.97%, 97.37%, 99.56%, and 99.75%, and a F1-Score of 99.98%, 97.69%, 98.89%, and 99.26% for the tested datasets. A comparison is presented in order to assess the performance of dDCA and the proposed model, along with state-of-the-art approaches for network anomaly detection. The proposed approach was able to surpass state-of-the-art approaches with the UNSW-NB15, and CSE-CIC-IDS2018 datasets, whereas the results obtained with NSL-KDD dataset are able to surpass a deep neural network based approach when measuring precision, recall, and F1-Score. The proposed approach aims to improve classification performance, as well as to propose a machine learning approach to the field of anomaly detection using bio-inspired models. The main challenges of the proposed model are, model dependence on certain parameters, such as the migration threshold for the DC population, the selection of segment size, as well as wavelet selection. The lack of multi-class classification, diminished performance when dealing with sparce or low footprint attacks, along with a further analysis of computational complexity are challenges presented as future work. Multiresolution analysis may provide insight to solve some of the mentioned challenges, such as multi-class classification. The segmented dDCA approach poses a lower computational complexity in comparison with the dDCA. However, the computational complexity added with the use of decision trees needs to be further analyzed. The proposed model may be adapted to the use of any MRA approach without decimation, such as the Empirical Mode Decomposition (EMD). Performance testing and comparison with MODWT is needed in order to demonstrate its effectiveness.

## Additional Information and Declarations

### Data Availability

The following information was supplied regarding data availability: The code of the results reported is available at GitHub: https://github.com/MR-Research/MRA-S-dDCA. Additional information: The UNSW-NB15 testing dataset is available at https://research.unsw.edu.au/projects/unsw-nb15-dataset. The NSL-KDD testing dataset is available at https://www.unb.ca/cic/datasets/nsl.html. The CIC-IDS2017 dataset and description can be found here: https://www.unb.ca/cic/datasets/ids-2017.html. The CSE-CIC-IDS2018 dataset description can be found here: https://registry.opendata.aws/cse-cic-ids2018/.
